# Editorial: Impact of viral infections on childhood asthma: susceptibility and pathomechanisms

**DOI:** 10.3389/falgy.2026.1916993

**Published:** 2026-07-15

**Authors:** Nicole Maison, Ulrich von Both, Jimmy Omony

**Affiliations:** 1 Department of Pulmonary and Allergy, Department of Pediatrics, Dr von Hauner Children’s Hospital, University Children's Hospital, Ludwig-Maximilians - University, Munich, Germany; 2 Comprehensive Pneumology Center (CPC-M), University of Munich, LMU Munich, German Center for Lung Research (DZL), Munich, Germany; 3 Institute of Asthma and Allergy Prevention (IAP), Helmholtz Munich – German Research Center for Environmental Health (GmbH), Munich, Germany; 4 Department of Infectious Diseases, Dr. von Hauner Children’s Hospital, LMU University Hospital, Ludwig-Maximilians-Universität München (LMU), Munich, Germany; 5 German Center for Infection Research (DZIF), Partner Site Munich, Munich, Germany

**Keywords:** allergic sensitization, asthma, epigenetics, epidemiology, RSV, viral infections

Childhood asthma remains one of the most common chronic diseases worldwide and is a major source of morbidity, healthcare utilization, and impaired quality of life (1, 2). Despite significant advances in our understanding of asthma pathogenesis, the mechanisms underlying the disease onset and progression remain poorly understood. Among the numerous factors implicated in asthma development, respiratory viral infections have emerged as critical contributors (3). Viruses such as respiratory syncytial virus (RSV), rhinovirus, seasonal influenza A/B, and other respiratory pathogens are well-known triggers of wheezing episodes and asthma exacerbations. Evidence increasingly suggests that viral infections which occur during critical periods of immune and lung development may have long-lasting effects on respiratory health (4, 5). However, the relationship between viral infections and childhood asthma is complex. It is influenced by genetic susceptibility, epigenetic regulation, environmental exposures, and allergic sensitization (2, 5).

A fundamental question in pediatric respiratory medicine is why certain children experience severe viral respiratory infections and long-term sequelae, whereas their peers recover without apparent consequences. To address this issue, Lu et al. investigated genetic susceptibility associated with RSV-related hospitalization in a cohort of Taiwanese children (Lu et al.). The authors identified genetic variants potentially associated with increased susceptibility to severe RSV infection requiring hospitalization. Although preliminary, these findings contribute to a growing body of evidence indicating that host genetic factors influence the clinical response to respiratory viral infections (6, 7). Understanding the genetic determinants of severe RSV disease may help identify children at elevated risk of adverse respiratory outcomes and provide insights into the biological pathways linking viral infection and asthma pathogenesis.

The importance of host susceptibility is intricately explored in the Perspective article by Pischedda et al., which examined whether early-life RSV infection may induce epigenetic changes that promote asthma development (Pischedda et al.). While epidemiological studies have consistently reported an association between severe RSV infection during infancy and later asthma, the mechanisms underlying this relationship remain controversial, thus warranting further investigation (4, 5). Pischedda et al. discuss emerging evidence of epigenetics suggesting that RSV infection may influence DNA methylation patterns, histone modifications, and non-coding RNA regulation, resulting in long-term alterations of immune responses and airway function. Such epigenetic modifications could provide a mechanistic bridge between transient viral exposure and persistent respiratory disease. Importantly, this perspective highlights the possibility that early-life infections occurring during critical developmental windows may leave a lasting biological imprint that shapes future susceptibility to asthma. The integration of epigenetic research into studies of respiratory disease offers promising opportunities to understand disease origins and identify novel preventive strategies, particularly in early childhood (8).

Together, the studies by Lu et al. and Pischedda et al. emphasize that the consequences of viral infections are determined not only by pathogen exposure but also by host-specific biological factors operating at multiple levels. Genetic predisposition may influence susceptibility to severe infection, while epigenetic responses may contribute to long-term alterations in airway and immune function.

Beyond disease inception, viral infections remain among the most important triggers of asthma morbidity in children (9). In this context, Jones et al. provide valuable epidemiological insights describing changing patterns of viral infection severity in a pediatric asthma cohort during the transition from COVID-19 lockdown measures to post-pandemic recovery (Jones et al.). Public health interventions implemented during the COVID-19 pandemic dramatically altered the circulation of common respiratory viruses, creating a unique opportunity to examine the relationship between viral exposure and asthma outcomes. The authors observed evolving patterns of viral infections and disease severity as community social restrictions were relaxed and viral circulation resumed. Their findings illustrate the profound influence that changes in viral epidemiology can have on children with asthma, which underscores the dynamic nature of respiratory disease risk. The study also highlights how population-level factors, including behavioral and environmental changes, may interact with individual susceptibility to shape asthma morbidity.

The observations reported by Jones et al. have broader implications for understanding asthma exacerbations. They reinforce the concept that respiratory viruses remain a major driver of acute disease burden and suggest that surveillance of viral circulation may play a vital role in predicting periods of increased risk for pediatric asthmatics. Furthermore, the study demonstrates how disruptions in viral exposure patterns can influence disease expression, offering new perspectives on the interactions between infection, immunity, and airway inflammation.

Although viral infections are central to asthma pathogenesis and exacerbation, they occur within a broader context of allergic and environmental influences. Hu et al. contribute to this understanding by characterizing allergen sensitization patterns among children with asthma in China in a retrospective cohort study (Hu et al.). The authors identified distinct allergen profiles and demonstrated considerable heterogeneity in sensitization patterns among pediatric patients. Although the study focused primarily on allergic factors rather than viral infections, its findings remain highly relevant to this Research Topic, since allergic sensitization is a key determinant of asthma phenotype and severity and may substantially influence the host response to respiratory viral infections. Previous studies have suggested that allergic airway inflammation can impair antiviral immune responses, potentially increasing susceptibility to virus-induced exacerbations. Conversely, viral infections may amplify preexisting allergic inflammation, creating a cycle that contributes to disease persistence and worsening symptoms (10).

The study by Hu et al., therefore, complements the viral-focused investigations presented in this collection by emphasizing the importance of considering asthma as a heterogeneous disease arising from multiple interacting biological pathways. Viral infections, allergic sensitization, and host susceptibility factors should not be perceived as independent determinants but rather as interconnected components of a complex disease network.

Taken together, these studies provide a multidimensional view of childhood asthma, highlighting viral infections as a central but interconnected factor. Genetic, epigenetic, environmental, and allergic influences all shape susceptibility, disease burden, and responses to infection, underscoring the need for an integrated approach spanning molecular mechanisms to population-based epidemiology.

Several important questions remain to be addressed regarding the link between viral infection and the development of pediatric asthma. Future longitudinal studies are needed to clarify the causal relationships between viral infections, epigenetic modifications, and asthma development. Larger and more diverse genetic studies may help identify robust susceptibility markers and uncover biological pathways amenable to intervention. Integrative approaches combining genomic, epigenomic, immunological, environmental, and clinical data will likely be necessary to fully characterize the mechanisms linking viral infections and asthma. Furthermore, recent advances in RSV prevention, including maternal immunization and long-acting monoclonal antibodies, provide unprecedented opportunities to evaluate whether reducing early-life viral disease can alter the trajectory of respiratory health and decrease the risk of subsequent asthma (11, 12).

In summary, the contributions assembled in this Research Topic highlight the role of viral infections in childhood asthma. By examining genetic susceptibility, epigenetic regulation, epidemiological trends, and allergic sensitization, these studies advance our understanding of the complex pathways connecting viral exposure to asthma development and exacerbation. Continued interdisciplinary research in this field will be essential for developing more effective strategies for prevention and management of pediatric asthma and ultimately reducing the global burden of the disease.
